# Dysfunctional LHX6 pallido-subthalamic projections mediate epileptic events in a mouse model of Leigh syndrome

**DOI:** 10.1172/JCI187571

**Published:** 2025-10-02

**Authors:** Laura Sánchez-Benito, Melania González-Torres, Irene Fernández-González, Laura Cutando, María Royo, Joan Compte, Miquel Vila, Sandra Jurado, Elisenda Sanz, Albert Quintana

**Affiliations:** 1 Institut de Neurociències, and; 2 Department of Cell Biology, Physiology and Immunology, Universitat Autònoma de Barcelona, Bellaterra, Barcelona Spain.; 3 Institute of Neuroscience, CSIC-UMH, Alicante, Spain.; 4 Neurodegenerative Diseases Research Group, Vall d’Hebron Research Institute-Network Center for Biomedical Research in Neurodegenerative Diseases (CIBERNED), Barcelona, Spain.; 5 Department of Biochemistry and Molecular Biology, Autonomous University of Barcelona, Barcelona, Spain.; 6 Catalan Institution for Research and Advanced Studies (ICREA), Barcelona, Spain.; 7 Focus Area for Human Metabolomics, Faculty of Natural and Agricultural Sciences, North-West University, Potchefstroom, South Africa.

**Keywords:** Inflammation, Neuroscience, Epilepsy, Mitochondria, Mouse models

## Abstract

Deficits in the mitochondrial energy–generating machinery cause mitochondrial disease, a group of untreatable and usually fatal disorders. Refractory epileptic events are a common neurological presentation of mitochondrial disease, including Leigh syndrome, a severe form of mitochondrial disease associated with epilepsy. However, the neuronal substrates and circuits for mitochondrial disease–induced epilepsy remain unclear. Here, using mouse models of Leigh syndrome that lack mitochondrial complex I subunit NDUFS4 in a constitutive or conditional manner, we demonstrated that mitochondrial dysfunction leads to a reduction of GABAergic neurons in the rostral external globus pallidus (GPe) and identified a specific affectation of pallidal *Lhx6*–expressing inhibitory neurons contributing to altered GPe excitability. Our findings revealed that viral vector–mediated *Ndufs4* reexpression in the GPe effectively prevented seizures and improved survival in the models. Additionally, we highlight the subthalamic nucleus (STN) as a critical structure in the neural circuit involved in mitochondrial epilepsy, as its inhibition effectively reduces epileptic events. Thus, we have identified a role for pallido-subthalamic projections in epilepsy development in the context of mitochondrial dysfunction. Our results suggest STN inhibition as a potential therapeutic intervention for refractory epilepsy in patients with mitochondrial disease, providing promising leads in the quest to identify effective treatments.

## Abstract 2

New research uncovers how faulty brain wiring drives mitochondrial epilepsy, proposing ways to stop seizures in otherwise untreatable childhood cases.

## Introduction

Mitochondrial diseases constitute a group of genetic disorders characterized by alterations in mitochondrial function, primarily leading to defective oxidative phosphorylation (OXPHOS), affecting 1 in 4,300 births (1). Mitochondrial diseases are often progressive, involving multiple organ systems. However, they primarily affect organs that rely on aerobic metabolism such as the CNS and muscular tissue. As a result, clinical manifestations are varied but typically feature prominent neurological and muscular components (2, 3).

Among the different signs, epilepsy stands out as one of the most prevalent CNS manifestations in mitochondrial disease. Epileptic seizures lead to rapid neuronal damage, characterized by histopathological findings such as microvacuolation, neuronal loss, eosinophilia, astrogliosis, and secondary myelin loss in affected patients (4). Reports indicate that epilepsy occurs in 35%–60% of individuals with biochemically confirmed mitochondrial disease (5, 6). Early-onset epilepsy is associated with reduced life expectancy in childhood, with a 50% mortality rate within the year following the onset of epilepsy (7). Notable differences in epilepsy prevalence exist among various mitochondrial diseases, and significant heterogeneity in the presentation of epileptic seizures among patients has been observed (8). These factors have contributed to our limited understanding of the pathogenic mechanisms underlying epilepsy in mitochondrial disorders.

Several hypotheses have been posited to link mitochondria dysfunction to epilepsy. For instance, energetic failure coupled with imbalances in calcium and ROS homeostasis could precipitate epileptic events, which in turn demand substantial energy. These events would subsequently exacerbate mitochondrial dysfunction, establishing a vicious cycle that ultimately leads to neuronal death (7–9). Alternatively, it has been proposed that the underlying pathophysiological process associated with mitochondrial epilepsies is initiated by the deficiency of the mitochondrial respiratory chain function in interneurons, which have been described as highly susceptible to OXPHOS deficiencies, especially those resulting from defects in complex I and complex IV (10–12). Thus, this would disrupt excitatory/inhibitory synaptic networks and promote neuronal hyperexcitability at the circuit level (13).

Several brain regions have been associated with epileptogenic circuitries, including the hippocampus; amygdala; frontal, temporal, and olfactory cortex; and basal ganglia (14, 15). However, the discrete areas involved in mitochondrial epilepsy remain unclear. Therefore, a deeper understanding of the anatomical basis of epilepsy is crucial for comprehending the origin of abnormal electrical activity, facilitating accurate diagnosis, and guiding appropriate management strategies.

In this regard, basal ganglia lesions are a hallmark of mitochondrial disease (16, 17), and the basal ganglia are believed to play a direct role in seizure generation, with multiple studies demonstrating their significant involvement in the propagation and control of various seizure types (14, 18–20). To explore the potential role of the basal ganglia in mitochondrial epilepsy, we dissected the epileptogenic circuit in an animal model of Leigh Syndrome, the most prevalent pediatric mitochondrial disease (21), which lacks mitochondrial complex I subunit NDUFS4 either ubiquitously or conditionally in GABAergic neurons (22–25).

## Results

### Restoration of Ndufs4 in external pallidal GAD2 neurons extends lifespan, reduces epileptic events, and abolishes local microglial reactivity in cKO mice.

**Figure 1:**
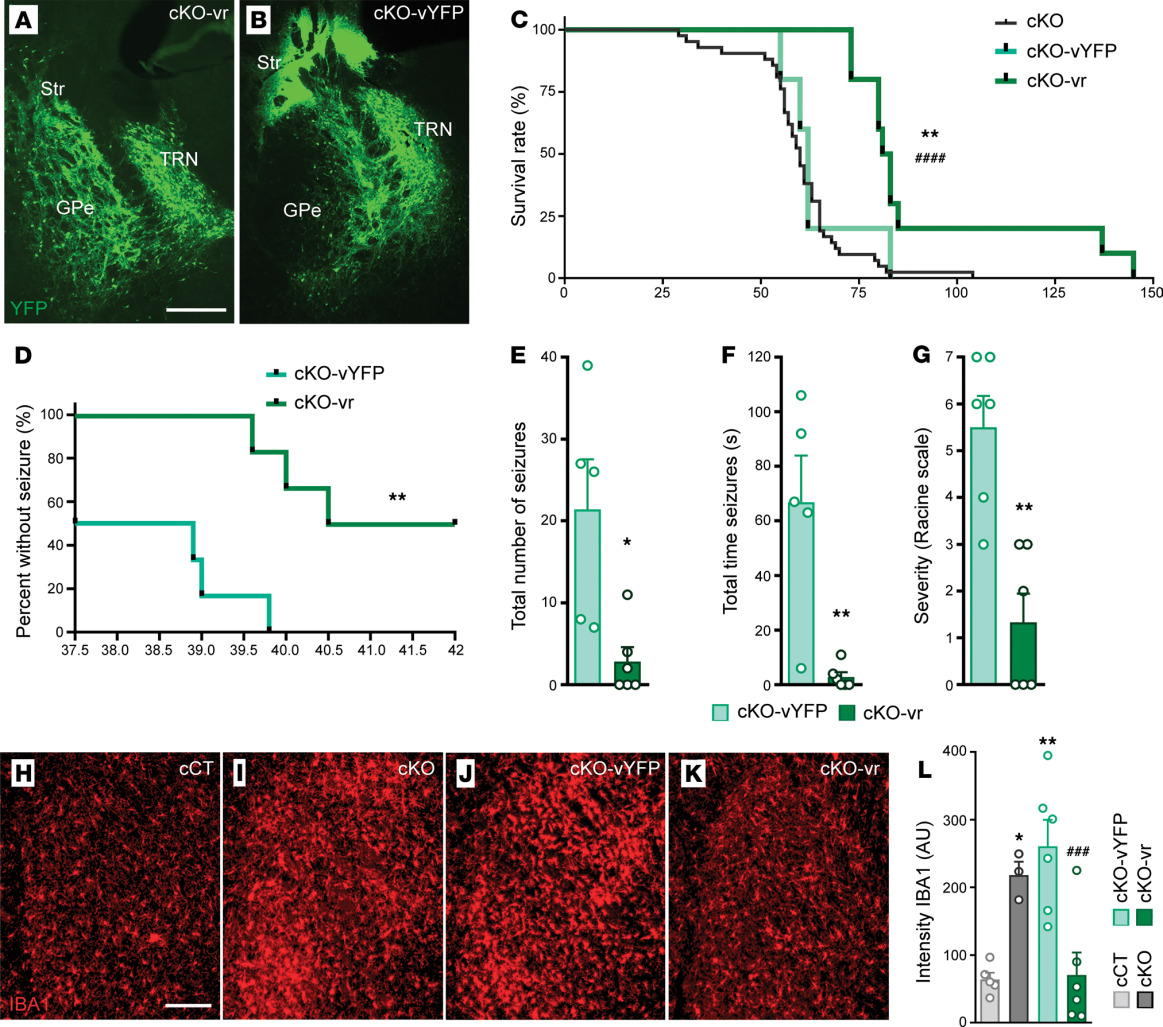
Reexpression of *Ndufs4* in *Gad2-*expressing GPe neurons reduces epilepsy and local microgliosis. (**A** and **B**) Representative images showing the injection site in the GPe of cKO-vr (**A**) and cKO-vYFP (**B**) mice. Scale bar: 400 μm. Str, striatum; GPe, external globus pallidus; TRN, thalamic reticular nucleus. The reduced transduction observed in the GPe of cKO-vYFP mice is likely due to neuronal death resulting from NDUFS4 deficiency, as shown in Figure 2. (**C**) Survival curves for cKO-vYFP (control group, *n* = 5), cKO-vr (rescue group, *n* = 7), and uninjected cKO mice (*n* = 42). **P < 0.01 indicates significant differences compared with cKO-vYFP group. ^####^*P* < 0.0001 indicates significant differences compared with the uninjected cKO group. Gehan-Breslow-Wilcoxon test. (**D**) Percentage of animals without epilepsy relative to body temperature. *n* = 6 per group. **P < 0.01, Gehan-Breslow-Wilcoxon test. (**E**) Total number of epileptic events during the induction protocol. cKO-vYFP mice *n* = 5, cKO-vr mice *n* = 6. *P < 0.05, Mann-Whitney *U* test. (**F**) Total duration of epileptic events. cKO-vYFP mice *n* = 5, cKO-vr mice *n* = 6. **P < 0.01, Mann-Whitney *U* test. (**G**) Maximum severity of epileptic events according to the modified Racine scale. *n* = 6 per group. **P < 0.01, unpaired 2-tailed *t* test. (**H**–**K**) Immunofluorescence staining for the microglial marker IBA1 (shown in red) in the GPe from cCT (**H**), cKO (**I**), cKO-vYFP (**J**), and cKO-vr (**K**) mice. Scale bar: 150 μm. (**L**) Quantification of IBA1 intensity, expressed in AU. cCT (*n* = 5), cKO (*n* = 3), cKO-vYFP (*n* = 6), cKO-vr (*n* = 6). *P < 0.05, **P < 0.01, indicates significant differences compared with cCT. ^###^*P* < 0.001, indicates significant differences compared with cKO-vYFP; 1-way ANOVA test.

Mice lacking the NDUFS4 subunit of mitochondrial complex I in *Gad2*-expressing GABAergic neurons (Gad2, Ndufs4c-KO, hereafter cKO mice) exhibit epileptic seizures, reduced lifespan, and glial reactivity in the external globus pallidus (GPe) within the basal ganglia (24, 25). Thus, we aimed to investigate the potential involvement of mitochondrial dysfunction in the GPe in the development of epilepsy by using viral vector–mediated stereotaxic restoration of NDUFS4 in this area (Supplemental Figure 1A; supplemental material available online with this article; https://doi.org/10.1172/JCI187571DS1). This approach successfully transduced both the GPe and the neighboring thalamic reticular nucleus (TRN), an area showing milder glial reactivity compared with the GPe (Supplemental Figure 1B), as described (24). Virus-mediated rescue of *Ndufs4* expression in cKO mice (cKO-vr mice, rescue group, Supplemental Figure 1C) significantly increased lifespan compared with those injected with the control viral vector (cKO-vYFP, Figure 1, A–C).

To study and compare both the frequency and severity of epileptic events between groups, seizures were induced by increasing the body temperature of cKO-vr and control mice (24, 26). Viral vector restoration of NDUFS4 in the GPe of cKO mice (cKO-vr group) led to a significant decrease in epileptic events compared with the control (cKO-vYFP) group (Figure 1, D–G). Of note, it was observed that a higher increase in body temperature was required for the rescued mice to develop epilepsy compared with the control group. Moreover, after the complete induction procedure, only 50% of the rescue group animals developed epilepsy compared with 100% of the control group animals (Figure 1D). Significant differences were found in the total number of epileptic events during the induction protocol (Figure 1E). Specifically, during the 10-minute prehabituation period, 50% of control-injected animals showed epileptic events but none of the viral rescue animals did (Figure 1, D and E). Differences in the number of epileptic events were also observed during the temperature increase period (Figure 1E). Likewise, significant reductions in the total duration and maximum severity of epileptic events were observed between the cKO-vr group and cKO-vYFP group (Figure 1, F and G).

cKO mice exhibit overt microglia/macrophage reactivity in the GPe (24). Therefore, we sought to determine whether *Ndufs4* reexpression could alter microglial/macrophage reactivity and infiltration in the GPe of cKO mice. The results of immunofluorescence analysis for the microglial protein IBA1 revealed that *Ndufs4* reexpression significantly reduced microglial reactivity in cKO mice compared with uninjected or control-injected animals (Figure 1, H–L).

To rule out the possibility that the beneficial effects of the rescue vector, compared with the control vector, were due to serotype-mediated differences in cellular transduction or distinct cellular compartmentalization of the control protein (mitochondrial or cytosolic), we generated a mitochondria-targeted control AAV5 vector by fusing the YFP sequence with the mitochondrial targeting sequence (MTS) for COX8 [AAV-DIO-COX8(MTS)·YFP; Supplemental Figure 1A]. Injection of this control viral vector, and the reexpression viral vector, into the GPe of cKO mice (cKO-vmtYFP and cKO-vr mice, respectively; Supplemental Figure 1, D–M) confirmed previous results, showing that *Ndufs4* reexpression in the GPe of cKO mice significantly increased their lifespan (Supplemental Figure 1N).

After demonstrating that restoring NDUFS4 in the GPe of cKO mice is crucial for improving their phenotype and reducing epileptic events in this animal model, we investigated whether the GPe was not only a necessary area involved in the epileptic process in cKO mice, but also sufficient for seizure generation. To that end, we stereotaxically injected viral vectors expressing either functional Cre recombinase (AAV-CRE·GFP) or a nonfunctional Cre as control (AAV-ΔCRE·GFP) (27–29) into the GPe of mice carrying 2 floxed *Ndufs4* alleles (*Ndufs4^fl/fl^* mice) (22). To assess the validity of this Cre-mediated approach, we generated a mouse line allowing selective expression of the ribosomal protein RPL22 tagged with HA upon Cre recombinase expression (RiboTag mice) (Ndufs4*^fl/fl^*-RiboTag), allowing the selective immunoprecipitation of ribosome-associated transcripts from transduced cells (30). Administration of AAV-CRE-GFP led to virtually absent *Ndufs4* expression in transduced cells, validating Cre recombinase activity (Supplemental Figure 2, A–D). Histological analysis of the injections confirmed that the expression of the viral vectors was restricted to the GPe and the TRN (Supplemental Figure 3, A and B). Neither of the mouse groups (injected with AAV-CRE·GFP or AAV-ΔCRE·GFP) showed spontaneous seizures or temperature-induced epileptic events, in contrast to cKO animals (Supplemental Figure 3C). This suggests that *Ndufs4* deletion limited to the GPe (and TRN) is not sufficient to trigger epilepsy or convulsive events. However, while *Ndufs4^fl/fl^* mice with specific deletion of *Ndufs4* in the GPe/TRN did not exhibit epileptic events, our results demonstrated that this deletion led to microgliosis confined to the injection site, a response absent in control mice (Supplemental Figure 3, D–J). This indicates that *Ndufs4* deletion in the GPe induces local microglial reactivity and/or macrophage infiltration.

### Lhx6-expressing GABAergic neurons in the GPe show increased vulnerability to mitochondrial dysfunction.

**Figure 2:**
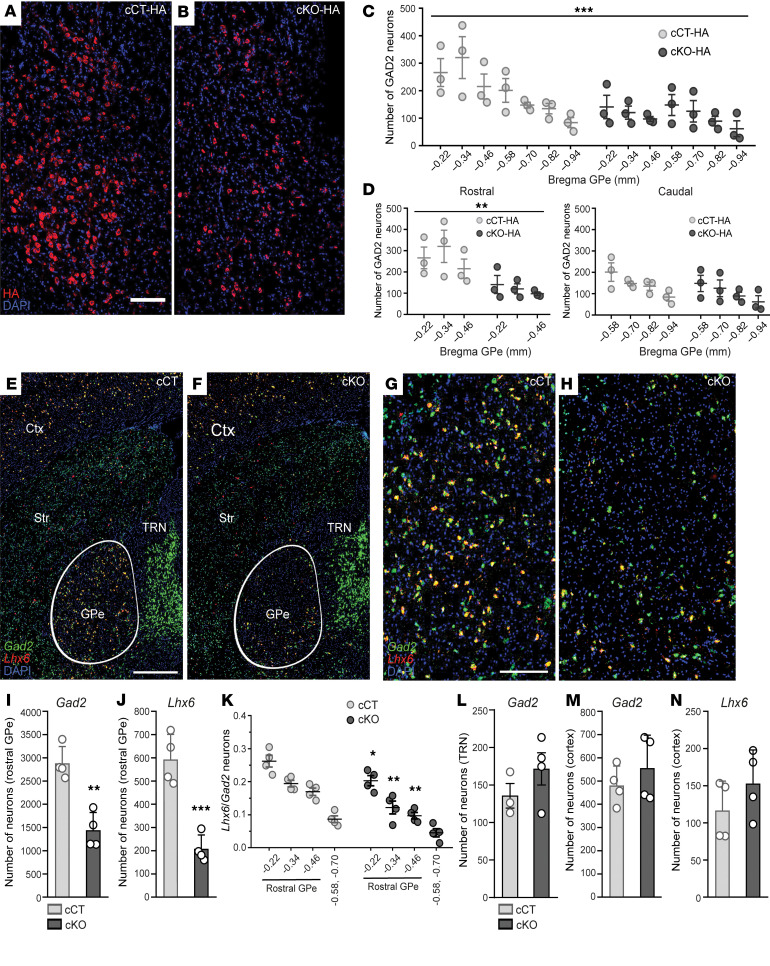
Loss of *Lhx6*-expressing GABAergic neurons in the rostral GPe of cKO mice. (**A** and **B**) Immunofluorescence analysis of GPe showing HA (red) staining in cCT-HA (**A**) and cKO-HA (**B**) mice. Scale bar: 200 μm. (**C**) Quantification of HA-positive (GAD2) neurons across GPe (from Bregma –0.22 mm to –0.94 mm) in cCT-HA and cKO-HA mice. *n* = 3 per group; 2-way ANOVA; ***P < 0.001 column factor effect (genotype). (**D**) Quantification of HA-positive (GAD2) neurons in rostral GPe (from Bregma –0.22 mm to –0.46 mm) and caudal GPe (from Bregma –0.46 mm to –0.94 mm) from cCT-HA and cKO-HA mice. *n* = 3 per group; 2-way ANOVA, **P < 0.01 column factor effect (genotype). (**E**–**H**) In situ hybridization analysis for *Gad2* (green) and *Lhx6* (red) mRNAs in brain sections containing rostral GPe of cCT (**E**) and cKO mice (**F**). Scale bar: 500 μm. Higher magnification images of rostral GPe of cCT (**G**) and cKO mice (**H**). Scale bar: 200 μm. (**I** and **J**) Quantification of total number of *Gad2*-expressing (**I**) and *Lhx6*-expressing (**J**) neurons in rostral GPe (from Bregma –0.22 mm to –0.46 mm) of cCT and cKO mice. *n* = 4 per group; **P < 0.01, ***P < 0.001, unpaired 2-tailed *t* test. (**K**) Proportion of *Lhx6*-expressing neurons relative to total number of *Gad2*-expressing neurons in rostral GPe of cCT and cKO mice. *n* = 4 for each group; 2-way repeat measures ANOVA followed by Šidák’s multiple-comparison test, *P < 0.05, **P < 0.01 column factor effect (genotype). (**L**) Quantification of total number of Gad2-expressing neurons in the TRN of cKO and cCT mice (from Bregma –0.22 mm to –0.46 mm). *n* = 3–4 per group. (**M** and **N**). Quantification of total number of *Gad2*-expressing (**L**) and *Lhx6*-expressing (**M**) neurons in primary somatosensory cortex of cCT and cKO mice (Bregma –0.34 mm) of cCT and cKO mice. *n* = 4 per group.

To assess the impact of *Ndufs4* deficiency on the abundance and survival of GABAergic neurons in the GPe, we generated a cKO mouse line expressing the RiboTag construct exclusively in *Gad2*-expressing GABAergic neurons (experimental mice, cKO-HA; control mice, cCT-HA), enabling the visualization of *Gad2* neurons with the use of an anti-HA antibody in cCT-HA and cKO-HA mice euthanized at P60 (Figure 2, A and B). A reduction in *Gad2*-expressing neurons was observed in the GPe of cKO-HA mice compared with cCT-HA mice (Figure 2C), particularly in the rostral part (from Bregma –0.22 mm to –0.46 mm, Figure 2D), underscoring the potential localization of a compromised neuronal population in this region.

Approximately 95% of GPe neurons are GABAergic and can be classified into different subtypes based on specific marker expression (31), each with distinct projections and anatomical distributions (31, 32). Notably, the *Lhx6*-expressing GABAergic subpopulation is more abundant in rostral GPe (32). In situ hybridization assays for *Gad2* and *Lhx6* on brain sections of cKO and cCT mice containing the GPe (Figure 2, E–H) showed a reduction in both *Gad2*-expressing neurons (Figure 2I) and *Lhx6*-expressing neurons (Figure 2J) in the rostral GPe in cKO mice compared with cCT mice. Moreover, the *Lhx6/Gad2* ratio was also reduced in cKO mice compared with cCT mice, suggesting increased vulnerability of *Lhx6* neurons in the rostral GPe to *Ndufs4* deficiency (Figure 2K). Neuronal counting showed a significant decrease in the neuronal populations in the rostral GPe of cKO mice compared with cCT mice, suggesting that reduced expression of *Gad2* and *Lhx6* was due to cell death rather than to loss of phenotypic markers (Supplemental Figure 4, A–C). In contrast to the GPe, no differences were observed in the number of *Gad2*-expressing neurons in the TRN of cKO mice (Figure 2, E, F, and L), as expected given the absence of *Lhx6-*expressing neurons in the TRN (33), thus confirming a primary role for the GPe in the phenotype of cKO mice. Additionally, reduction of *Gad2-* or *Lhx6*-expressing neurons was not observed in the cortex of these mice (Figure 2, M and N), an area with abundant *Gad2-* and *Lhx6*-expressing neurons, ruling out a global affectation of *Lhx6*-expressing neuronal populations in cKO mice.

### Fatal epileptic events lead to activation of the subthalamic nucleus in cKO mice.

**Figure 3:**
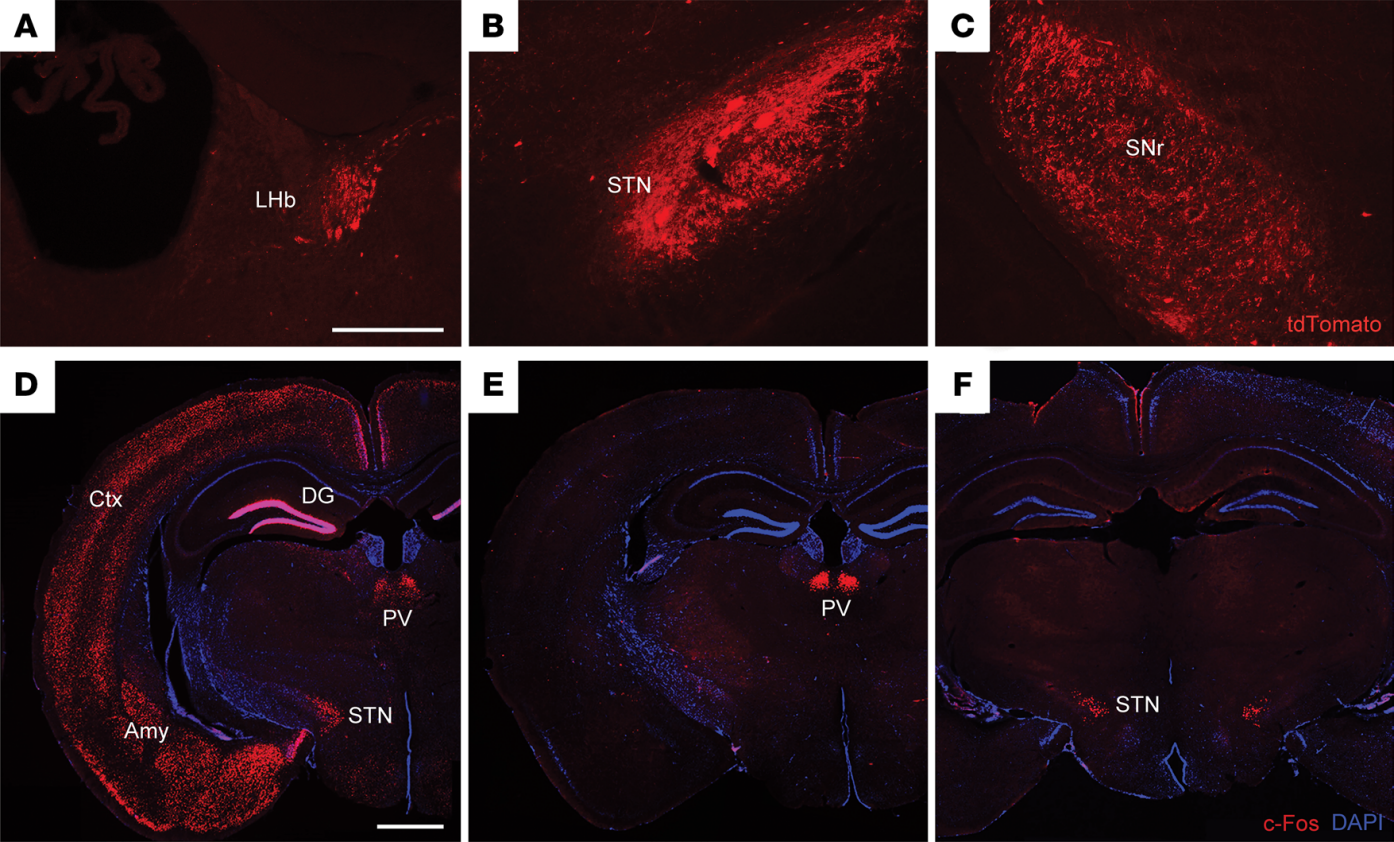
Activation of the STN during fatal epileptic events. (**A**–**C**) TdTomato labeling of brain areas receiving inputs from the GPe: lateral habenula (LHb) (**A**), subthalamic nucleus (STN) (**B**), and substantia nigra pars reticulata (SNr) (**C**). Scale bar: 300 μm. *n* = 3 per group. (**D**–**F**) Representative images of the brain areas in cKO animals showing c-Fos immunoreactivity after fatal epileptic events. (**D**) Generalized c-Fos expression pattern. (**E**) Restricted c-Fos expression in the paraventricular thalamus (PV). (**F**) Restricted c-Fos expression in the subthalamic nucleus (STN). DG, dentate gyrus; Amy, amygdala; Ctx, cortex. Scale bar: 1 mm.

The GPe exhibits predominant projections to various basal ganglia regions, including the striatum, subthalamic nucleus (STN), internal globus pallidus, and substantia nigra pars reticulata. In addition, GPe projections also extend to the TRN, cerebral cortex, amygdala, and lateral habenula (31). To specifically identify projections from the rostral GPe, stereotaxic injections of a Cre-expressing viral vector (AAV1-CRE·GFP) were administered in the rostral GPe of tdTomato reporter mice (Ai9 mice) (34). This confirmed pallidal projections to the lateral habenula, STN, and substantia nigra pars reticulata (Figure 3, A–C).

Furthermore, to elucidate the putatively affected and/or involved regions within the epileptogenic circuit of cKO mice, we assessed c-Fos protein expression as a proxy for neuronal activation after epileptic events (35, 36). Using this approach, we identified two distinct patterns of neuronal activation in response to fatal epileptic events in cKO animals. Approximately half of the cKO mice exhibited widespread c-Fos staining across various brain regions, including the dentate gyrus of the hippocampal formation, cerebral cortex, STN, and paraventricular thalamus, among others (Figure 3D). In contrast, a second group of cKO animals showed c-Fos staining confined to the paraventricular thalamus and STN regions only (Figure 3, E and F). Thus, despite the marked difference in overall c-Fos activation, the STN and the paraventricular thalamus were the only areas consistently activated across all animals.

### Excitatory/inhibitory tone imbalance in the GPe of cKO mice.

**Figure 4:**
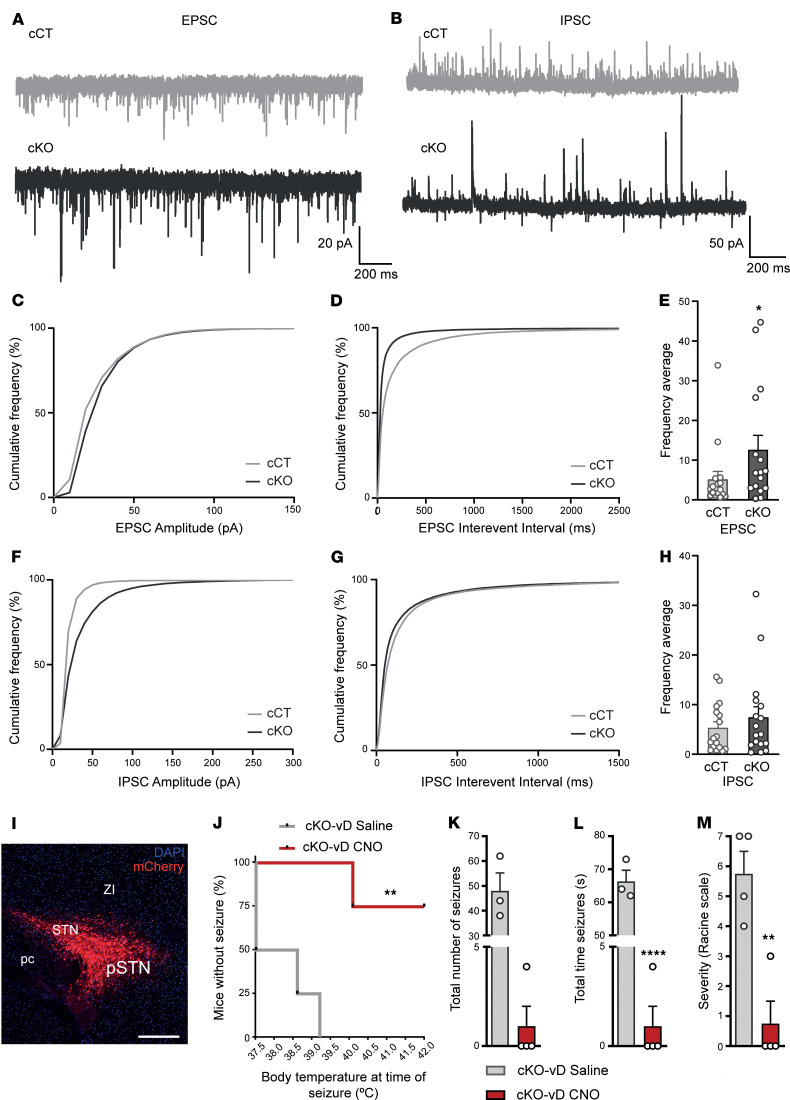
Altered pallido-subthalamic projections participate in the development of seizures in cKO mice. (**A**–**H**) Spontaneous inhibitory and excitatory postsynaptic currents in GPe-containing brain slices from cCT and cKO mice (*n* = 17 cells, 6 mice per group). (**A** and **B**) Representative traces showing EPSCs (**A**) and IPSCs (**B**) recorded at -70 mV and 0 mV, respectively. (**C**) Cumulative frequency plots of EPSC amplitudes. ****P < 0.0001, Kolmogorov–Smirnov test. (**D**) Cumulative distribution of EPSC interevent intervals. ****P < 0.0001, Kolmogorov–Smirnov test. (**E**) Average frequency of EPSCs. *P < 0.05, Mann-Whitney test. (**F**) Cumulative frequency plots of IPSC amplitudes. ****P < 0.0001, Kolmogorov–Smirnov test. (**G**) Cumulative distribution of IPSC interevent intervals. ****P < 0.0001, Kolmogorov–Smirnov test. (**H**) Average frequency of IPSCs. (**I**–**M**) Analysis of temperature-induced epilepsy in cKO mice comparing the group with chemogenetic inhibition of the STN (cKO-vD CNO) with the control group (cKO-vD saline). (**I**) Representative image of the stereotaxic injection site of the AAV8/2-CaMKIIa-hM4Di·mCherry viral vector in the STN. Scale bar: 400 μm. STN, subthalamic nucleus; pSTN, parasubthalamic nucleus; pc, cerebellar peduncle; ZI, zona incerta. (**J**) Percentage of animals without epilepsy relative to body temperature. A value less than 100% at the initial temperature (37.5°C) indicates that the mouse experienced epileptic events during the habituation period. *n* = 4 per group. **P < 0.01, log-rank test (Mantel-Cox). (**K**) Total number of events during the induction protocol. cKO-vD CNO *n* = 4; cKO-vD saline *n* = 3. *P* = 0.057, Mann-Whitney *U* test. (**L**) Duration of epileptic events. cKO-vD CNO *n* = 4; cKO-vD saline *n* = 3. ****P < 0.0001, unpaired 2-tailed *t* test. (**M**) Maximum severity of epileptic events according to the Racine scale. *n* = 4 per group. **P < 0.01, unpaired 2-tailed *t* test.

Pallidal *Lhx6*-expressing GABAergic neurons represent one of the GPe subpopulations that project to the glutamatergic neuronal populations in the STN (32, 37). In turn, the STN provides the main excitatory input onto the GPe (31). Therefore, we hypothesized that loss of *Lhx6*-expressing neurons in the GPe could imbalance the GPe-STN loop and alter the inhibitory-excitatory balance in the GPe. To that end, we recorded spontaneous excitatory and inhibitory postsynaptic currents (sEPSCs and sIPSCs) in brain slices from cCT cKO mice (Figure 4, A–H, and Supplemental Figure 5). Our results underscored a significant increase in the event amplitude of both sEPSCs (Figure 4C) and sIPSCs (Figure 4F) in cKO neurons compared with controls and an increase in the number of large sIPSC events (Supplemental Figure 5). Regarding event frequency, a significant shift in the cumulative distribution of sEPSC interevent intervals was observed in cKO neurons compared with cCT neurons (Figure 4D), indicating a decrease in interevent intervals and therefore an increase in event frequency. This was further supported by the significantly higher average frequency of sEPSCs in cKO neurons compared with cCT neurons (Figure 4E).

In contrast, GPe neurons in cKO mice presented a slight reduction in the cumulative distribution of sIPSC interevent intervals (Figure 4G) without a significant change in average sIPSC frequency (Figure 4H) compared with cCT neurons. Taken together, these results suggest that the loss of *Lhx6*-positive neurons in the GPe leads to a broad alteration of both excitatory and inhibitory synaptic activity, likely reflecting both an increase in STN excitatory inputs and compensatory reorganization of the local GPe circuitry.

### Chemogenetic inhibition of glutamatergic neurons in the STN attenuates temperature-induced epilepsy in cKO mice.

Given the increase in STN activity observed in cKO mice, we hypothesized that inhibiting the STN could mitigate epileptic events in cKO mice.

To achieve chemogenic inhibition of glutamatergic neurons in the STN of cKO mice, an AAV8/2-CaMKIIa-hM4Di·mCherry viral vector was stereotaxically administered into the STN of cKO mice (Figure 4I). Subsequently, to assess the role of the STN in epileptic events after chemogenetic inhibition, mice were subjected to a thermally induced epileptogenic paradigm (24) on P54. After completing the entire induction procedure, only 25% of animals with chemogenetic inhibition (treated with clozapine N-oxide [CNO]) exhibited epilepsy, in contrast to 100% in the saline group (Figure 4J). In addition, the CNO-treated mice presented a higher threshold for temperature-induced epileptic events, together with a decrease in duration and maximum severity of epileptic events during the induction protocol, compared with the saline group (Figure 4, K–M).

### Specific loss of Lhx6-expressing GABAergic neurons in the GPe is conserved in animals constitutively lacking NDUFS4.

**Figure 5:**
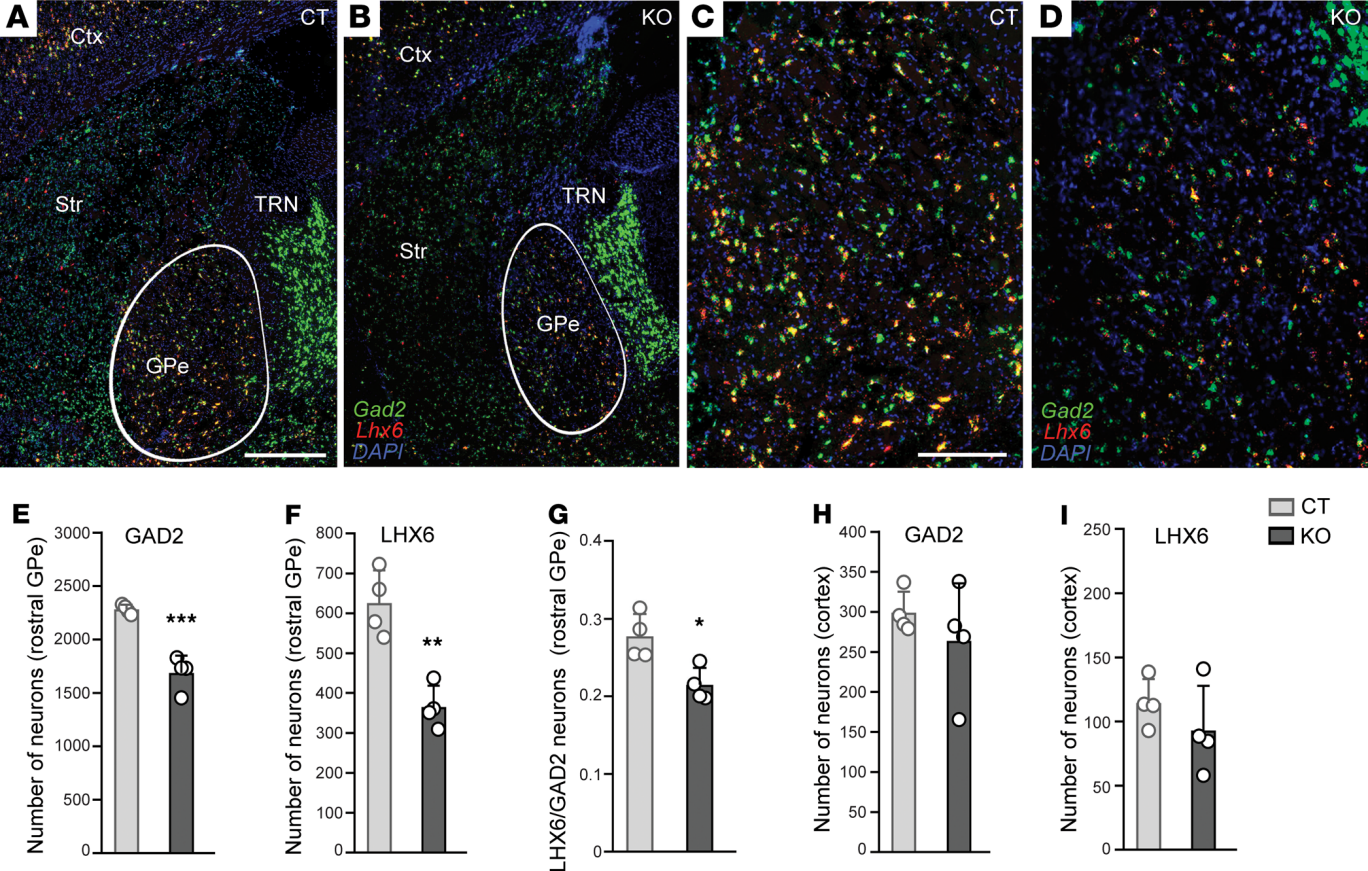
Reduced number of *Gad2-* and *Lhx6-*expressing neurons in the rostral GPe of KO mice. (**A**–**D**) In situ hybridization analysis for *Gad2* (green) and *Lhx6* (red) mRNAs in brain sections containing the rostral GPe of CT (**A** and **C**) and KO (**B** and **D**) mice. (**C** and **D**) show magnified views of the GPe in **A** and **B**, respectively. (**E** and **F**) Quantification of the total number of *Gad2*-expressing (**A**) and *Lhx6*-expressing (**B**) neurons in the rostral GPe of Ndufs4-KO and control mice (from Bregma –0.22 mm to –0.46 mm). *n* = 4 per group. **P < 0.01, ***P < 0.001, unpaired 2-tailed *t* test. (**G**) Proportion of *Lhx6*-expressing neurons relative to the total number of *Gad2*-expressing neurons in the rostral GPe of CT and KO mice. *n* = 4 per group. *P < 0.05, unpaired 2-tailed *t* test. (**H** and **I**) Quantification of the total number of *Gad2*-expressing (**H**) and *Lhx6*-expressing (**I**) neurons in the primary somatosensory cortex of CT and KO mice (Bregma –0.34 mm). *n* = 4 per group.

cKO mice manifest an overt vulnerability of GABAergic neurons in the rostral GPe to *Ndufs4* deletion, particularly within the *Gad2*/*Lhx6*-expressing subpopulation. Thus, we aimed at validating whether this vulnerability is conserved in animals that constitutively lack NDUFS4 (KO mice), an established model of Leigh syndrome (22, 23, 29, 38). In agreement with our results in the conditional *Ndufs4* KO in GABAergic neurons, in situ hybridization assays showed a reduction in the number of *Gad2*-expressing neurons in the rostral GPe in the constitutive KO group compared with controls (Figure 5, A–E). Furthermore, a decrease in the number of *Lhx6*-expressing neurons in the rostral GPe was also observed in the KO mice compared with controls (Figure 5, A–D, and F). A significant reduction in the *Lhx6*/*Gad2* ratio further underscored a preferential decrease in *Lhx6*-expressing neurons in the rostral GPe in animals lacking *Ndufs4* compared with control mice (Figure 5G). However, no such difference in the number of *Gad2-* or *Lhx6*-expressing neurons was observed in the cortex (Figure 5, H and I), an area not predominantly affected in KO mice (22, 23, 29, 38). These results suggest that the reduction of *Lhx6*-expressing neurons in the rostral GPe is a conserved response to *Ndufs4* deficiency, highlighting the pallidal region’s specific vulnerability.

### NDUFS4 restoration in external pallidal GABAergic neurons reduces temperature-induced seizures and extends lifespan in KO mice.

**Figure 6:**
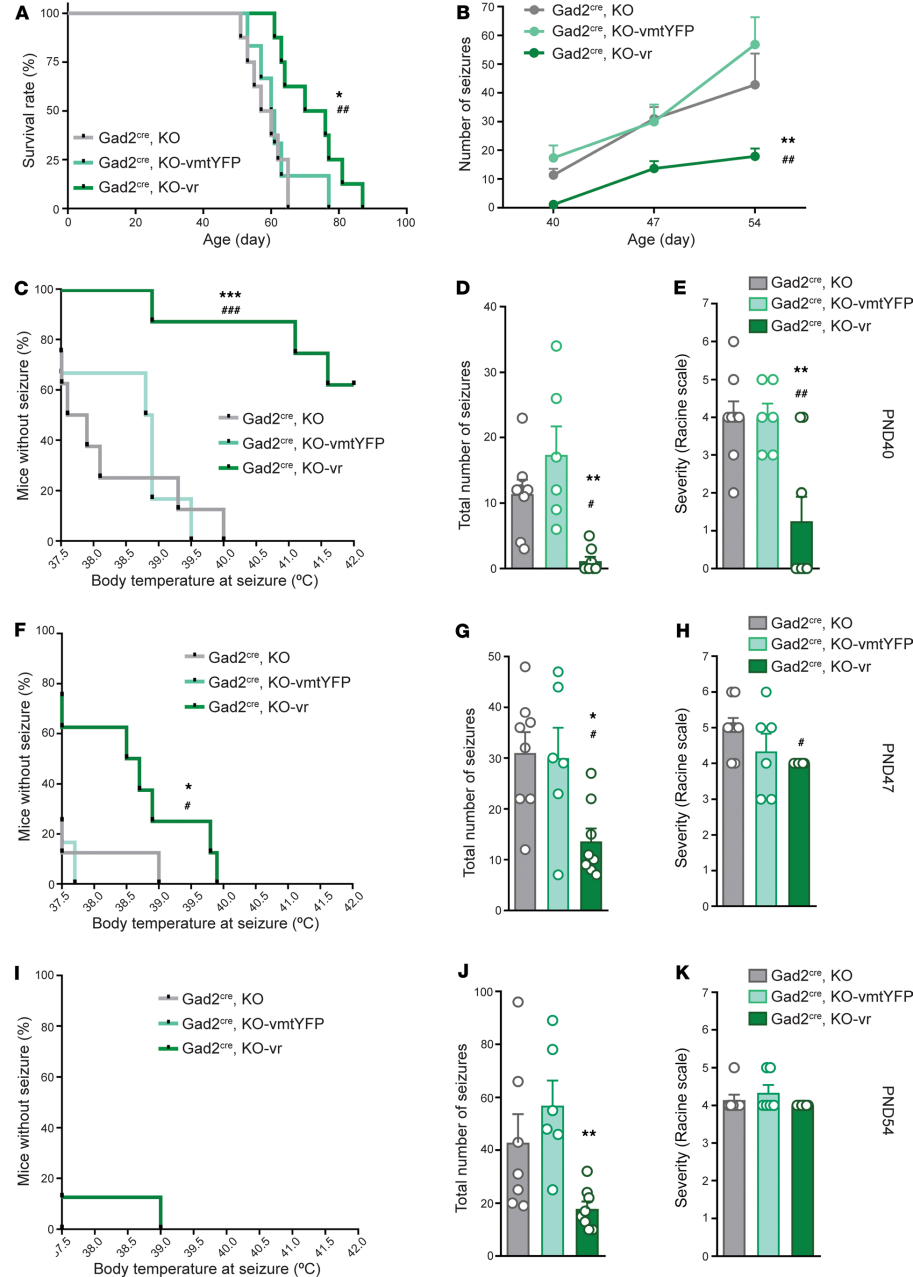
*Ndufs4* reexpression in external-pallidal *Gad2*-expressing neurons extends lifespan and reduces temperature-induced seizures in *Ndufs4*-deficient animals. (**A**) Survival curves of Gad2^Cre^-KO (uninjected; *n* = 8), Gad2^Cre^-KO-vmtYFP (control-injected; *n* = 6), and Gad2^Cre^-KO-vr (viral rescue; *n* = 8) mice. ^##^*P* < 0.01 indicates significant differences compared with Gad2^Cre^-KO, *P < 0.05 indicates significant differences compared with Gad2^Cre^-KO-vmtYFP, log-rank test (Mantel-Cox). (**B**) Temporal progression of total epileptic events during inductions at P40, P47, and P54 in Gad2^Cre^-KO (*n* = 7–8), Gad2^Cre^-KO-vmtYFP (*n* = 6), and Gad2^Cre^-KO-vr (*n* = 8). Mixed-effects analysis followed by Tukey’s multiple-comparison test: ^##^*P* < 0.01 indicates significant differences compared with Gad2^Cre^-KO, **P < 0.01 indicates significant differences compared with Gad2^Cre^-KO-vmtYFP. (**C**–**E**) Effect of NDUFS4 restoration in GPe on temperature-induced epilepsy in Gad2^Cre^-KO mice at P40 assessed by percentage of animals remaining seizure-free after increasing body temperature (**C**), total number of epileptic events during induction protocol (**D**), and severity of epileptic events according to Racine scale (**E**). Gad2^Cre^-KO *n* = 8; Gad2^Cre^-KO-vmtYFP *n* = 6; Gad2^Cre^-KO-vr *n* = 8. **P < 0.01, ***P < 0.001, indicates significant differences compared with KO-vmtYFP. ^#^*P* < 0.05, ^##^*P* < 0.01, ^###^*P* < 0.001, indicates significant differences compared with Gad2^Cre^-KO. Signficance was determined by log-rank test (Mantel-Cox) (**C**), Kruskal-Wallis test (**D**), and 1-way ANOVA (**E**). (**F**–**H**) Effect of NDUFS4 restoration in GPe on temperature-induced epilepsy in Gad2^Cre^-KO mice at P47 assessed by percentage of animals remaining seizure-free after increasing body temperature (**F**), total number of epileptic events during induction protocol (**G**), and severity of epileptic events according to Racine scale (**H**). Gad2^Cre^-KO *n* = 8; Gad2^Cre^-KO-vmtYFP *n* = 6; Gad2^Cre^-KO-vr *n* = 8. *P < 0.05, indicates significant differences compared with KO-vmtYFP. ^#^*P* < 0.05, indicates significant differences compared with Gad2^Cre^-KO. Significance was determined by Gehan-Breslow-Wilcoxon test (**F**), 1-way ANOVA followed by Tukey’s multiple-comparison test (**G**), and Kruskal-Wallis followed by Dunn’s multiple-comparison test (**H**). (**I** and **K**) Effect of NDUFS4 restoration in GPe on temperature-induced epilepsy in Gad2^Cre^-KO mice at P54 assessed by percentage of animals remaining seizure-free after increasing body temperature (**I**), total number of epileptic events during induction protocol (**J**), and severity of epileptic events according to Racine scale (**K**). Gad2^Cre^-KO *n* = 7; KO-vmtYFP *n* = 6; Gad2^Cre^-KO-vr *n* = 8. **P < 0.01, indicates significant differences compared with Gad2^Cre^-KO-vmtYFP. Significance was determined by log-rank test (Mantel-Cox) (**I**), Kruskal-Wallis test followed by Dunn’s multiple-comparison test (**J**), and 1-way ANOVA followed by Tukey’s multiple-comparison test (**K**).

KO mice exhibit a fatal phenotype associated with reduced lifespan (approximately between P50 and P60) (23, 29). KO mice manifest spontaneous epilepsies (23) and are susceptible to thermally induced seizures (39). Thus, we sought to assess the involvement of GPe GABAergic populations in the development of the epileptic phenotype of KO mice. To that end, we first generated a mouse line with a constitutive deletion of *Ndufs4* and concurrent expression of Cre recombinase in GABAergic neurons (Gad2^Cre^-KO mice). These mice subsequently received a stereotaxic injection of a Cre-dependent AAV expressing either *Ndufs4* or a mitochondria-targeted YFP in the GPe. Reexpression of *Ndufs4* in the GPe increased the survival of the Gad2^Cre^-KO mice (Gad2^Cre^-KO-vr versus Gad2^Cre^-KO-vmtYFP group and noninjected Gad2^Cre^-KO mice) (Figure 6A). Analysis of thermally induced epileptic events during the induction protocols at different disease stages (P40, P47, and P54) in Gad2^Cre^-KO mice revealed an overall significant decrease in seizure frequency in the Gad2^Cre^-KO-vr group compared with the Gad2^Cre^-KO-vmtYFP and noninjected Gad2^Cre^-KO control groups (Figure 6B). It was noteworthy that we observed a differential impact of NDUFS4 restoration during disease progression (Figure 6, C–K). In this regard, at P40, Gad2^Cre^-KO-vr mice presented a higher resistance to thermally induced seizures, showing approximately a 3-fold reduction in the number of animals developing seizures compared with Gad2^Cre^-KO-vmtYFP or Gad2^Cre^-KO mice (Figure 6C). In addition, a reduction in the frequency (Figure 6D) and severity (Figure 6E) of epileptic events was observed in the Gad2^Cre^-KO-vr group. At P47, Gad2^Cre^-KO-vr mice continued to exhibit resistance to epilepsy induction compared with the Gad2^Cre^-KO-vmtYFP and Gad2^Cre^-KO groups. However, all animals eventually experienced epileptic events (Figure 6F). Likewise, a significant decrease was observed in the total number of epileptic events during the induction protocol in the Gad2^Cre^-KO-vr group compared with both control groups (Figure 6G). However, no differences were noted in the maximum severity of these events (Figure 6H). Finally, at a late stage of the disease, specifically at P54 (Figure 6, I–K), the Gad2^Cre^-KO-vr group continued to show a significant reduction in the total number of epileptic events compared with the Gad2^Cre^-KO-vmtYFP and Gad2^Cre^-KO control groups (Figure 6J). Nonetheless, no differences were found in the percentage of mice developing seizures (Figure 6I) or the severity of the events (Figure 6K).

## Discussion

Epilepsy significantly affects quality of life, and early-onset cases are associated with a severe prognosis and reduced life expectancy (7, 40). Epilepsy is a frequent manifestation in mitochondrial disease, a group of severe and untreatable pathologies resulting from mutations affecting mitochondrial function. However, there are no effective therapies for mitochondrial epilepsies, as they are often resistant to common antiepileptic drugs (41).

The underlying mechanisms of mitochondrial epilepsy remain unclear. Variability in seizure incidence and prevalence across mitochondrial disease has hindered the study of the underpinnings of epilepsy in mitochondrial disease (7, 8, 12). In this regard, the generation of animal models of mitochondrial disease developing epilepsy (24, 39, 42, 43) has paved the way to characterize the mechanisms involved in mitochondrial epilepsy. Among these models, studies in a mouse model of Leigh syndrome lacking the complex I subunit NDUFS4 have been instrumental in identifying the involvement of GABAergic neurons in the development of epileptic events (24, 25). However, the circuitry involved in mitochondrial epilepsy remains to be elucidated. Here, we used these validated mouse models to identify the critical role of a pallido-subthalamic circuitry in the mitigation of mitochondrial epilepsy.

Leigh syndrome is characterized by overt symmetrical lesions in the brainstem and basal ganglia (44), areas particularly vulnerable to *Ndufs4* deficiency in mice (24, 29). Electrophysiological alterations in GPe neurons have been shown to precede epileptic events in cKO (Ndufs4-cKO) mice (24). Our results show that *Ndufs4* reexpression in the GPe reduces microglial reactivity and improves the epileptic phenotype observed in both Ndufs4-cKO and KO (Ndufs4-KO) mice. These results highlight that the regulation of epileptic events by the GPe is not restricted to a conditional imbalance of the excitatory/inhibitory inputs, potentially intensifying the epileptic condition (13). Conversely, selective deletion of *Ndufs4* in the GPe increases local microglial reactivity but is not sufficient to elicit the appearance of epileptic seizures. One possible explanation for the lack of epileptic phenotype is that GPe dysfunction due to *Ndufs4* deletion requires targeting of a larger population of GPe neurons than what is achieved through *Ndufs4* reexpression in rescue experiments. However, this seems unlikely given the broad targeting and the overt microglial/macrophage response observed after *Ndufs4* deletion in the GPe. Thus, our results seem to underscore a role for the GPe in the limitation of the propagation of epileptic events initiated elsewhere. The specific area or anatomical region where epilepsies originate from mitochondrial dysfunction remains unknown. Fos gene product staining studies in Ndufs4-cKO mice have revealed neuronal activation in the cortex, dentate gyrus of the hippocampal formation, and the amygdala, among other areas associated with epilepsy in humans and rodents (45–48). However, these events may be attributed to synchronization and excitotoxicity associated with epilepsy (49), precluding their direct implication in epilepsy genesis (25).

Basal ganglia circuits have been suggested to prevent cortical seizure propagation through feedback mechanisms (14, 50, 51). Accordingly, Ndufs4-cKO mice present increased cortical and hippocampal activity initiated by impaired interneuron function (25). Thus, our results suggest that the inhibitory network of basal ganglia is compromised in Ndufs4-cKO mice, impairing the control of excitatory neuron activity and leading to the development of epilepsy. In this context, rescuing NDUFS4 in the GPe could potentially restore basal ganglia circuit function, ultimately improving the epileptic phenotype.

To our knowledge, our study has identified for the first time the selective loss of *Gad2* neurons in the rostral segment of the GPe in Ndufs4-cKO mice, in particular *Lhx6-*expressing neurons, which have been described to be abundant in this area (31, 32). Deletion of *Lhx6* has been associated with impaired cortical interneuron migration and differentiation and seizures (52–54). Thus, it is possible that lack of NDUFS4 may lead to widespread interneuron development, in line with reports suggesting neurodevelopmental alterations in mitochondrial disease models (55, 56). However, no alterations in the numbers of either cortical *Gad2*- or *Lhx6*-expressing interneurons were observed, in line with previous reports (25) ruling out a global developmental defect in interneuron populations as the cause for the seizures in the Ndufs4-KO model, underscoring the phenotypic diversity of mitochondrial pathologies. In this regard, the epileptic phenotype commonly worsens as disease progresses in individuals with Leigh syndrome (57) and in NDUFS4-KO mice (23, 24, 39). Thus, we hypothesize that progressive degeneration of GABAergic populations in affected areas, such as the GPe, reduces the overall inhibitory tone, with potential implications for epilepsy-associated neurodegeneration in both humans and rodents (58, 59).

Our activity-mapping studies highlighted a consistent activation of both the STN and paraventricular thalamus after fatal epileptic events. In mammals, the paraventricular thalamus functions as a stress sensor, activated by physical or psychological stressors (60–62), suggesting it is a proxy of the stressful response triggered by the epileptic event. In contrast, the STN receives major connections from the GPe (63, 64) given that the *Lhx6*-expressing neurons are one of its main inputs (37). In keeping with this, our electrophysiological recordings of the GPe in cKO (Ndufs4-cKO) mice suggest a general imbalance in synaptic activity, with an overall increase in excitatory inputs. Given the GPe’s predominant GABAergic projections to the STN, this imbalance would likely result in functional disinhibition of the STN, which under conditions of heightened excitatory drive is prone to generating pathological rhythmic bursting. Such bursting has been previously observed in basal ganglia–thalamocortical circuitopathies, including those seen in Parkinson’s disease and epilepsy (65, 66).

However, the interpretation of increased excitatory drive must be tempered by the potential limitations imposed by mitochondrial dysfunction. Prior studies have shown that impaired mitochondrial function can reduce the intrinsic firing capacity or disrupt the autonomous pace making of GPe neurons (67, 68). Thus, it remains an open question whether the GPe in this model exhibits a net increase or decrease in effective output. One possibility is that despite increased synaptic excitation, GPe neurons may fail to sustain firing or lose synchronization, ultimately leading to reduced inhibitory output to the STN.

A net reduction in GPe output, whether due to neuronal loss, desynchronization, or energy failure, would relieve tonic inhibition from the STN, thereby enhancing its excitability. In turn, the STN provides glutamatergic excitatory projections to both the internal globus pallidus/ substantia nigra pars reticulata and thalamic relay nuclei (63, 64), potentially amplifying activity throughout the basal ganglia–thalamocortical loop. Notably, thalamic relay neurons and the TRN are key structures in the generation and maintenance of spike-and-wave discharges characteristic of absence seizures (69, 70). In this framework, STN-driven excitation of thalamic circuits could entrain burst firing in relay neurons and disrupt thalamic activity, contributing to the emergence of pathological oscillations. Additionally, the STN serves as an input nucleus of the basal ganglia through the hyper-direct pathway, receiving direct glutamatergic connections from motor, premotor, and frontal cortical areas (71) and establishing reciprocal connections with various thalamic nuclei and the prefrontal cortex (72). In addition, abnormal STN activation may lead to increased general excitation of corticothalamic loops, leading to secondary damage in downstream brainstem and corticothalamic nuclei, contributing to the pathology (73, 74).

Although we did not directly record from the STN in this study, our findings in the GPe support a mechanistic model in which circuit-level disinhibition of the STN initiates or sustains rhythmic bursting patterns that propagate through the basal ganglia–thalamocortical circuitry. Supporting this, chemogenetic inhibition of glutamatergic STN neurons is sufficient to reduce the frequency and severity of thermal-induced epileptic events in Ndufs4-cKO mice. Collectively, these findings implicate the STN as a critical GPe projection involved in controlling and propagating epilepsies in the context of mitochondrial dysfunction.

Neuromodulation of the STN, such as by deep brain stimulation, has been shown to significantly alter epileptic activity (75) in patients with drug-resistant epilepsy as well as in murine experimental models (75–80). Although deep brain stimulation can induce activation or inhibition of the targeted area (81), our results suggest that inhibiting the STN may be beneficial for controlling epilepsy in patients with mitochondrial disease. Based on our results, we propose that the loss of LHX6 neurons in the GPe leads to reduced inhibitory control in the STN, contributing to the propagation of epilepsy in Ndufs4-cKO mice.

In conclusion, this work underscores a role for pallido-subthalamic projections in the development of epilepsy in the context of mitochondrial dysfunction, suggesting STN inhibition as a potential therapeutic intervention for refractory epilepsy in patients with mitochondrial disease.

## Methods

### Sex as a biological variable.

Our study examined male and female animals, and similar findings are reported for both sexes.

### Mice.

Gad2^Cre^ (*Gad2*-IRES-Cre) mice (82) were procured from The Jackson Laboratory (stock 028867). *Ndufs4*^lox/lox^ and Ndufs4^Δ/+^ mice (The Jackson Laboratory, stock 027058) were previously generated by our group (22, 23). Both male and female mice of various ages were used in this study. All mice underwent a minimum of 10 generations of backcrossing and were on a C57BL/6J background.

Mice with a conditional deletion of *Ndusf4* in *Gad2*-expressing GABAergic neurons (Gad2^Cre^; Ndufs4^lox/lox^, Ndufs4-cKO, or cKO mice) were obtained by crossing mice with one floxed *Ndufs4* allele (23) (The Jackson Laboratory, stock 026963) and expressing Cre recombinase under the control of the *Gad2* promoter (Gad2^Cre^; Ndufs4^lox/+^) with mice carrying 2 floxed *Ndufs4* alleles (Ndufs4^lox/lox^). Littermate controls were Gad2^Cre^; Ndufs4^lox/+^ (Ndufs4cCT, or cCT) mice. *Ndufs4*^fl/fl^-RiboTag mice (*Ndufs4*^lox/lox^ Rpl22^HA/HA^) were generated by crossing mice with one floxed *Ndufs4* allele (*Ndufs4*^lox/+^) with RiboTag mice (Rpl22^HA/HA^), which express a Cre-dependent HA-tagged ribosomal protein Rpl22 (30) (The Jackson Laboratory, stock 011029). In all cases, the genotype of the offspring was determined by PCR analysis using primer sequences previously described (22, 24). For labeling studies, tdTomato reporter mice (Ai9, The Jackson Laboratory, stock 007909) (34) were used.

Mice were group-housed under a 12-hour light/12-hour dark cycle at 22°C with ad libitum access to rodent chow (Teklad Global Rodent Diet, Envigo, 2014S) and water, unless otherwise specified. Sex- and age-balanced groups of 2- to 7-month-old mice were used for all experimental procedures, and no sex differences were observed. After surgeries, animals were individually housed until the completion of all experimental procedures. Sample sizes were determined using power analyses, and the number of animals per group in each experiment (*n*) is provided in figure legends.

### Viral vector production.

Recombinant AAVs were generated in HEK293T cells with AAV1 or AAV5 coat serotype. Purification involved sucrose and CsCl gradient centrifugations, and the final suspension was prepared in 1× HBSS at a titer of 2 × 10^9^ viral genomes/μL, following established protocols (29). The AAV preparations were aliquoted and stored at –80°C until stereotaxic injection. AAV8/2-CamKII-hM4i-mCherry vector (Addgene plasmid 50477) was a gift from A. Ranson, Universitat Autònoma de Barcelona, Bellaterra, Barcelona, Spain.

### Stereotaxic surgery.

All surgical procedures were conducted under aseptic conditions. Animal anesthesia was induced and maintained with 5% and 1%–1.5% isoflurane/O_2_, respectively. Analgesia (5 mg/kg ketoprofen; Sanofi-Aventis) and ocular protective gel (Viscotears, Bausch + Lomb) were administered. Mice were then positioned on a heating pad within a robot-operated 3D (stereotaxic) frame (Neurostar) for intracerebral viral vector delivery. Stereotaxic coordinates were normalized using a correction factor (Bregma-Lambda distance/4.21) based on Paxinos and Franklin’s coordinates (83). AAV preparations were bilaterally delivered into the GPe using the following coordinates: anteroposterior –0.46 mm from Bregma; mediolateral ± 2.00 mm; and dorsoventral –4.00 mm from the skull surface. The injection rate was maintained at 0.1 μL/min (0.5 μL per injection site) using a 5 μL syringe (Hamilton). AAV preparations were bilaterally delivered into the STN using the following coordinates: anteroposterior –1.94 mm from Bregma; mediolateral ± 1.50 mm; and dorsoventral –4.50 mm from the skull surface. The injection rate was maintained at 0.1 μL/min (0.5 μL per injection site) using a 5 μL syringe (Hamilton). After infusion, the needle remained in place for 6 minutes to allow proper diffusion. Subsequent needle withdrawal was performed at 1 mm/min to minimize off-target viral leakage. Only animals with accurate targeting were included in the experiment.

### Epilepsy induction.

Mouse body temperature was regulated using a rectal temperature probe and a heat lamp connected to a feedback-controlled temperature system (Physitemp Instruments). In brief, following the method outlined by Oakley et al. (26), body temperature was incrementally raised by 0.5°C every 2 minutes until a seizure was induced or a maximum temperature of 42°C was reached. Subsequently, mice were cooled using a small fan. To achieve chemogenetic STN neuronal silencing, CNO was administered at 1 mg/kg (i.p.) as described (84).

### Brain slice preparation.

Coronal brain slices containing the GPe (280 μm thick) were obtained from 1-month-old male mice (P30–P40). Deeply anesthetized mice (i.p. injection) were transcardially perfused with cold-cutting solution containing (mM) 200 sucrose, 26 NaHCO_3_, 10 glucose, 3 KCl, 1.25 NaH_2_PO_4_, 4 MgSO_4_, and 0.5 CaCl_2_. The same solution was used during slicing in a Leica VT1000S vibratome. After sectioning, slices were placed in a holding chamber containing standard artificial cerebrospinal fluid solution (mM): 124 NaCl, 26 NaHCO_3_, 10 glucose, 3 KCl, 1.25 NaH_2_PO_4_, 1 MgSO_4_, and 2 CaCl_2_, and then stored at room temperature (22°C–24°C). After an incubation period of at least 30 minutes, slices were transferred to a submersion recording chamber at room temperature. Incubation solutions were continuously bubbled with a gas mixture of 95% O_2_/5% CO_2_.

### Electrophysiology.

The recording chamber was perfused with artificial cerebrospinal fluid solution gassed with 5% CO_2_/95% O_2_. Whole-cell voltage-clamp recordings were obtained from WT and cKO animals. Patch recording pipettes (5–7 MΩ) were filled with a solution containing 115 mM CsMeSO_3_, 20 mM CsCl, 10 mM Hepes, 2.5 mM MgCl_2_, 4 mM Na_2_-ATP, 0.4 mM Na-GTP, 10 mM sodium phosphocreatine, 0.6 mM EGTA, pH 7.25, osmolarity 290 mOsm. Neurons in the GPe were voltage-clamped at –70 mV to record EPSCs or at 0 mV to record IPSCs.

Electrophysiological recordings and data acquisition were performed with a Multiclamp 700B amplifier, a 1550B digitizer, and pClamp 10 software (Molecular Devices). Recorded traces were analyzed using Clampfit 10 (Molecular Devices). All the recordings were performed using transmitted light illumination under a microscope equipped with a 40× water immersion objective.

### Tissue punch preparation.

For protein and gene expression analysis, mice were euthanized 3 weeks after surgery. Brains were rapidly extracted and fresh-frozen on dry ice. Coronal brain sections (300 μm thick) were obtained using a cryostat, covering the region from Bregma –0.10 mm to –1.34mm (83). The GPe was bilaterally dissected using a 2 mm micro-punch and stored at –80°C until further use.

### Western blot analysis.

To validate NDUFS4 reexpression, GPe punches from Ndufs4-KO mice injected with AAV5-DIO-vmtNDUFS4 or AAV5-DIO-vmtYP were homogenized in SDS sample buffer containing 62.5 mM Tris-HCl (pH 6.8; Merck Millipore, 648311 and 109063), 2% SDS (Thermo Fisher Scientific, BP166), and 10% glycerol (Sigma-Aldrich, G7757). Protein concentrations were determined using the Pierce BCA Protein assay kit (Thermo Fisher Scientific, 23225) following the manufacturer’s protocol. Prior to heat-denaturation, DTT (50 mM; MilliporeSigma, 646563) and bromophenol blue (0.1% w/v; MilliporeSigma, B5525) were added to 20 μg of protein lysates.

Samples were separated by 4%–20% gradient SDS-PAGE (Bio-Rad, 4561094) and transferred to a nitrocellulose membrane using the Trans-Blot Turbo RTA Mini Nitrocellulose Transfer kit (Bio-Rad, 1704270). Membranes were blocked for 1 hour in 5% (w/v) dried skimmed milk in Tris-buffered saline with 0.1% Tween-20 (TBS-T), followed by overnight incubation at 4°C with primary antibodies against NDUFS4 (1:1,000; Invitrogen, PA5-21677) and β-tubulin (1:10,000; MilliporeSigma, T7816). The next day, membranes were washed 3 times in TBST-T and incubated with the corresponding HRP-conjugated secondary antibodies (1:10,000; Jackson ImmunoResearch, 111-035-003, 115-035-003). After additional washes in TBS-T, protein bands were visualized using Clarity Western ECL Substrate (Bio-Rad, 1705060) and imaged with the Chemidoc MP Imaging System (Bio-Rad).

### RiboTag and gene expression assays.

To isolate polysome-associated mRNAs from *Ndufs4*-deficient cells, GPe punches from WT-RiboTag and Ndufs4*^fl/fl^*-RiboTag mice injected with AAV1-CRE·GFP were homogenized in 400 μL of buffer as previously described (85). After centrifugation, 20% of the cleared lysate was saved as input, while the remaining fraction was incubated with 2 μL of anti-HA antibody (BioLegend, 415930) for 4 hours at 4°C in rotation. Immunoprecipitation was performed by adding 100 μL of protein A/G magnetic beads (Thermo Fisher Scientific, 88803) to the antibody-conjugated samples, followed by O/N incubation at 4°C with rotation.

The following day, immunoprecipitates were washed 3 times in high-salt buffer, and RNA was extracted from input and immunoprecipitate samples using the RNeasy Micro kit (QIAGEN, 74104) with DNase I treatment. RNA quantification was performed using the Quant-iT RiboGreen RNA assay kit according to the manufacturer’s instructions (Invitrogen, Thermo Fisher Scientific, R11490).

For gene expression analysis, qRT-PCR assays were conducted by using the Luna Universal Probe One-Step RT-qPCR kit (New England BioLabs, 174E3006L). A total of 1 ng RNA per sample was analyzed, and relative expression levels were calculated using the standard curve method (85) and normalized to Gapdh. Amplification efficiency (80%–120%) and linearity (*r²* ≥ 0.99) were confirmed using the AriaMx Real-Time PCR System (Agilent Technologies). *Gapdh* and *Ndufs4* mRNA levels were detected using specific TaqMan assays (Mm99999915_g1 for Gapdh, Mm00656176_m1 for Ndufs4; Thermo Fisher Scientific); Cre expression was determined using a custom-made probe (CTGCCACCAGCCAGCTAT, GGGCACTGTGTCCAGACC, CCCTGGAAGGGATTTTTGAAGCAA).

### Neuronal counting.

To quantify the number of neurons in the rostral GPe, brains of Ndufs4cCT (control) and Ndufs4-cKO (experimental group) were collected. Mouse brains were fixed in 4% paraformaldehyde dissolved in PBS. After dehydration with ethanol, the paraffin blocks including 2 brains from both genotypes were sectioned into 5 μm coronal slices from Bregma +0.02 mm to –0.94 mm. Tissue sections were deparaffined with xylene 3 times for 10 minutes, rehydrated using an ethanol gradient (100% 2 washes, 95% 2 washes, 70% and 50% for 5 minutes each wash step) and tap water, and stained with Harris Hematoxylin (Sigma-Aldrich, HHS16) for 10 minutes. After subsequent dehydration using graded ethanol and xylene, slices were sealed with DPX mounting medium.

Brain sections were scanned with an Olympus VS200 slide scanner and further analyzed using Imaris software (version 10.2, Oxford Instruments). Cell counting was carried out in rostral sections from the GPe, from –0.22 mm to –0.46 mm (*n* = 5 per group) using Artificial Intelligence Trainable Segmentation (Machine Learning) to distinguish between tissue background and cells. Total cell numbers were obtained, and neurons were distinguished from other cell types by size (area in μm^2^ < 15 = other cell types, area in μm^2^ > 15 = neurons) and curated in a blinded manner to ensure the accuracy and consistency of the automated cell counting.

### Tissue processing and immunofluorescence analysis.

Upon CO_2_ asphyxiation, mouse brains were promptly dissected. The collected brains were fixed overnight in 4% paraformaldehyde dissolved in PBS and subsequently cryoprotected using 30% sucrose in PBS. Cryosectioning was performed by freezing the brains for 5 minutes in dry ice, followed by sectioning using a freezing microtome. For immunofluorescence, 30 μm free-floating sections for AAV targeting and c-Fos and microgliosis analysis or 20 μm free-floating sections for HA staining and neuron count were treated with a blocking solution containing 10% normal donkey serum (NDS) and 0.2% Triton X-100 in PBS for 1 hour at room temperature. Subsequently, the sections were incubated overnight at 4°C with a primary antibody solution in PBS containing 0.2% Triton X-100 and 1% NDS. The solution included the following antibodies: anti-GFP (Abcam, ab13970) at 1:2,000; anti-HA (Thermo Fisher Scientific, 71-5500) at 1:1,000; anti-IBA1 (Abcam, ab178846) at 1:1,000; anti-TOM20 (Santa Cruz Biotechnology, sc-11415) at 1:750; and anti-cFOS (Santa Cruz Biotechnology, sc-7202) at 1:750. After 3 washes in PBS with 0.2% Triton X-100, the sections were exposed to a secondary antibody solution containing the secondary antibody of interest (1:500; Abcam, ab150173; Invitrogen, Thermo Fisher Scientific, A21206 and A31572; Thermo Fisher Scientific, A21207) for 1 hour at room temperature. After incubation, sections underwent three 5-minute washes in PBS with 0.2% Triton X-100 and were mounted on slides using DAPI Fluoromount (Electron Microscopy Sciences, 17984-24) before visualization with an EVOS imaging system (Thermo Fisher Scientific) or a Zeiss LSM 700 tracking confocal microscope. Microgliosis was analyzed using ImageJ (NIH) software (Fiji, version 1.52). To determine the number of GABAergic neurons in the GPe, Ndufs4cCT-HA (cCT-HA) mice (control group) and Ndufs4-cKO-HA (cKO-HA) mice (experimental group) were used. This approach allowed the labeling of GABAergic GAD2 cells in the area of interest using an anti-HA antibody, as described in the previous section. To obtain representative images of the entire GPe, 7 sections of 20 μm thickness were selected at intervals of 100 μm, spanning a total of 0.84 mm within the area of interest (from Bregma –0.22 mm to –1.06 mm). The Zeiss LSM 700 confocal microscope equipped with a ×20 objective was used to capture images. A total of 15 images were acquired, with an interval of 1.34 μm between optical sections within the 20 μm thick section. To cover the entire GPe area, 3 to 5 images (depending on the position in Bregma) were taken with a minimum overlap of 10%, and subsequently, a 3D collage was created using the Grid Stitching function and the linear blend fusion method in ImageJ (NIH) software (86). These collages contained information on the DAPI signal (corresponding to nuclear labeling) and HA signal (for GAD2 neuron labeling) from the 15 optical sections taken. The counting of GPe cells was performed blindly using Imaris software (version 9.2, Oxford Instruments). The surface mask function with 3D images was used to automate cell counting based on HA labeling with the following settings: maximum cell size of 15 μm (to differentiate closely spaced cells) and minimum cell size of 5 μm (to exclude incomplete cell fragments or nonspecific labeling). The mask generated for each cell with these settings had to include DAPI within its boundaries to be counted as valid. The results obtained were manually reviewed to ensure the accuracy and consistency of the automated counting.

### In situ hybridization assays.

Mouse brains were snap-frozen in Tissue-Tek OCT compound (Sakura) with dry ice and stored at –80°C until cryosectioning. Coronal sections (16 μm) containing the rostral GPe were used for RNAscope analysis (Advanced Cell Diagnostics), following the manufacturer’s instructions. The following probes were employed: Mm-Gad2 (Advanced Cell Diagnostics, 439371) and Mm-Lhx6-C2 (Advanced Cell Diagnostics, 422791-C2). All in situ hybridization assays were imaged using an Olympus VS200 ASW scanner and analyzed with QuPath open-source software. Cell counting was conducted in GPe rostral sections from Bregma –0.22 mm to –0.46 mm in 3–4 slices/animal, bilaterally (*n* = 3 animals). The number of *Gad2*- and *Lhx6*-expressing cells was determined using QuPath (87).

### Statistics.

Data are presented as the mean ± SEM. Statistical analyses were performed using GraphPad Prism v9.0 software. Appropriate tests were selected based on the experimental design, as specified in the figure legends. Statistical significance (*P* < 0.05) is indicated in the figure legends. The number of mice (*n*) refers to the number of animals per group in each experiment. Different cohorts of mice were used for different tests to avoid repeated testing, and no attrition was observed.

### Study approval.

The present studies in animals were reviewed and approved by the Universitat Autònoma de Barcelona Ethics Committee (Bellaterra, Spain) and the Generalitat de Catalunya (Barcelona, Spain).

### Data availability.

Data are available in the Supporting Data Values file.

## Author contributions

LSB designed research studies, conducted experiments, acquired data, analyzed data, and wrote the manuscript. MGT, IFG, LC, MR, and JC conducted experiments, acquired data, and analyzed data. MV and SJ designed research studies, analyzed data, and provided reagents. ES and AQ designed research studies, analyzed data, provided reagents, and wrote the manuscript.

## Funding support

This work is the result of NIH funding, in whole or in part, and is subject to the NIH Public Access Policy. Through acceptance of this federal funding, the NIH has been given a right to make the work publicly available in PubMed Central.

- Two Ministerio de Economía y Competitividad Ramon y Cajal fellowships (RyC2019-028501-I to ES and RyC-2012-11873 to AQ).
- A Ministerio de Ciencia e Innovación (MICINN) Ramon y Cajal fellowship (RYC2022-037332-I to LC).
- Two predoctoral fellowships from Ministerio de Ciencia, Innovación y Universidades (MICIU) (PRE2018-083179 to LSB and PRE2020-094293 to IFG).
- An MICIU predoctoral fellowship (FPU19/03990 to MGT).
- ES was supported by MICIU Proyectos I+D+i “Retos Investigacion” (RTI2018-101838-J-I00, to ES) and Ministerio de Ciencia e Innovación (PID2019-107633RB-I00 and PID2022-142544OB-I00).
- AQ received funds from the European Research Council (starting grant 1178 NEUROMITO, ERC-2014-StG-638106), SAF2017-88108-R, PID2020-114977RB-I00, PID2023-151947OB-I00, RED2022-134786-T, RED2024-154130-T, 381180 funded by MICIU/AEI/10.13039/5011000110330 and by MICIU/AEI /10.13039/501100011033 1181 and Next GenerationEU/PRTR,2 AGAUR (2017SGR-323, 2021SGR-720), Fundació TV3-La 1182 Marató (202030), and “la Caixa” Foundation (ID 100010434), under the agreement 1183 LCF/PR/HR20/52400018.
- AQ is a recipient of an Institució Catalana de Recerca i Estudis Avançats Academia award.

## Supplementary Material

Supplemental data

Unedited blot and gel images

Supporting data values

## Version 1 10/02/2025

In-Press Preview

## Version 2 12/01/2025

Electronic publication
