# Supplementary material for: Dysfunctional LHX6 pallido-subthalamic projections mediate epileptic events in a mouse model of Leigh syndrome
Source: J Clin Invest. 2025 Oct 2;135(23):e187571. doi: 10.1172/JCI187571 (PMC12646652; doi:10.1172/JCI187571)
Supplement: Supplemental data [file jci-135-187571-s150.pdf]

Supplemental data

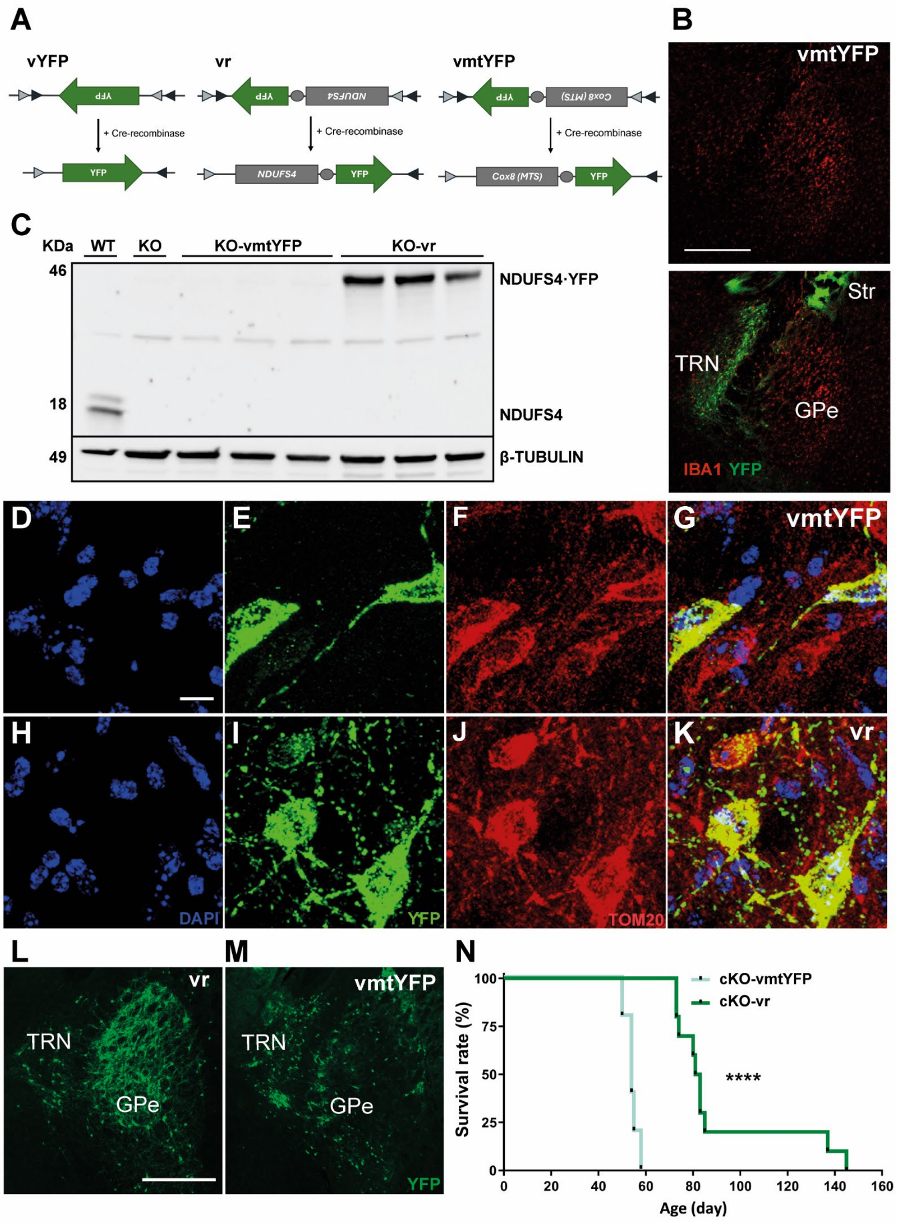

Figure S1. Validation of the mitochondria-targeted control viral vector and confirmation of lifespan extension following Ndufs4 re-expression in the GPe of cKO mice. A) Schematic representation of AAVs used to induce YFP, NDUFS4·YFP or MTS·YFP expression specifically in Cre-expressing cells. **B)** Immunofluorescence staining in brain sections containing the GPe and RTN from vmtYFP-injected cKO. Images show IBA1 staining alone and the merged signal of IBA1 with YFP expression. Scale bar = 500 µm. **C)** Immunoblot analysis of NDUFS4 and β-TUBULIN protein levels in vmtYFP- and vr-injected mice (n = 3). NDUFS4 migrates at ~18 KDa and NDUFS4·YFP migrates at ~49 kDa. A wild-type and an Ndufs4KO sample were included in the first and second lanes as positive and negative controls, respectively. **D-K)** Immunofluorescence analysis of cKO mice following stereotactic injection into the GPe with either the AAV5-DIO-COX8(MTS)·YFP viral vector (cKO-vmtYFP mice; D-G) or the AAV5-DIO-NDUFS4·YFP viral vector (cKO-vr mice; H-K). Panels show: nuclei stained with DAPI **(D,H),** YFP **(E, I)** and TOM20 **(F, J)** staining. An overlay of all stainings shows

mitochondrial localization of vmtYFP **(G)** and NDUFS4·YFP **(K).** Scale bar = 10 µm. L-N) *Ndufs4* re-expression in the GPe extends the lifespan of cKO Mice. Images show stereotactic injection into the GPe of the rescue AAV5-DIO-NDUFS4·YFP **(L)** or control AAV5-DIO-COX8(MTS)·YFP **(M)** viral vectors. Graph **(K)** shows lifespan extension in cKO-vr mice (n=10) compared to cKO-vmtYFP mice (n=5). RTN: Reticular thalamic nucleus, GPe: Globus pallidus externus. Scale bar = 400 µm. **** p<0.0001, Log-rank test (Mantel-Cox).

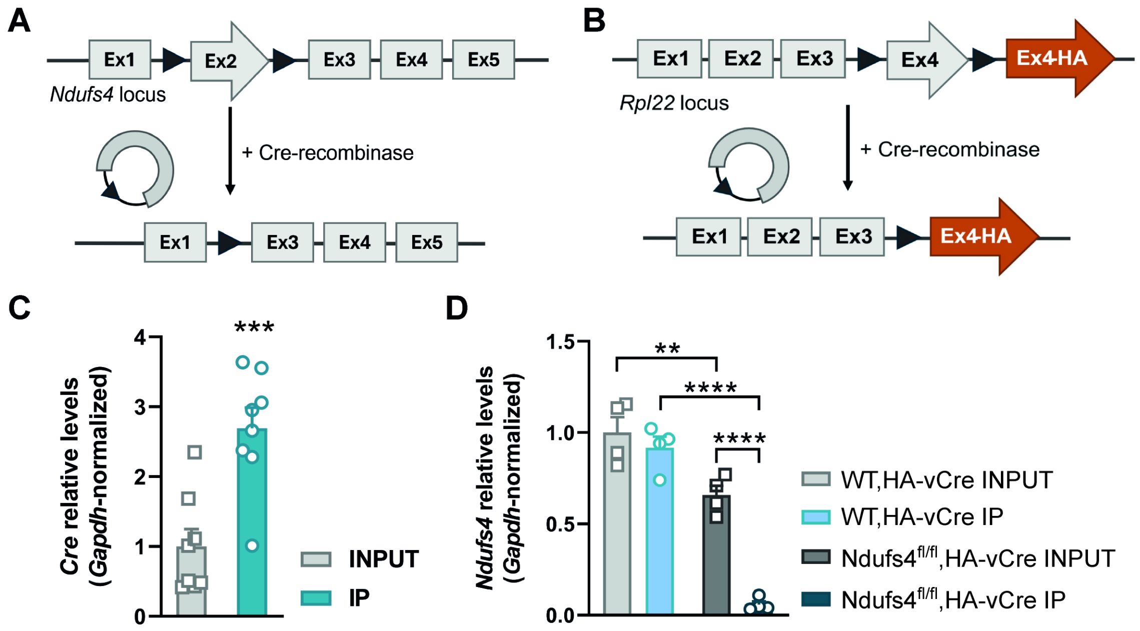

Figure S2. Validation of *Ndufs4* deletion in Cre-expressing cells. A) Genetic approach to achieve Cre-dependent *Ndufs4* deletion in the GPe. **B)** Genetic strategy to obtain Cre-dependent expression of hemagglutinin (HA)-tagged ribosomes. INPUT: whole-tissue GPe sample. IP: immunoprecipitated sample (CRE-expressing cells) **C)** *Cre* mRNA levels relative to Gapdh (n=8 per group). *** p<0.001, unpaired t-test. **D)** *Ndufs4* mRNA levels relative to Gapdh (n=4 per group). Two-way ANOVA followed by Tukey's multiple comparisons test. ** p<0.01, **** p<0.0001

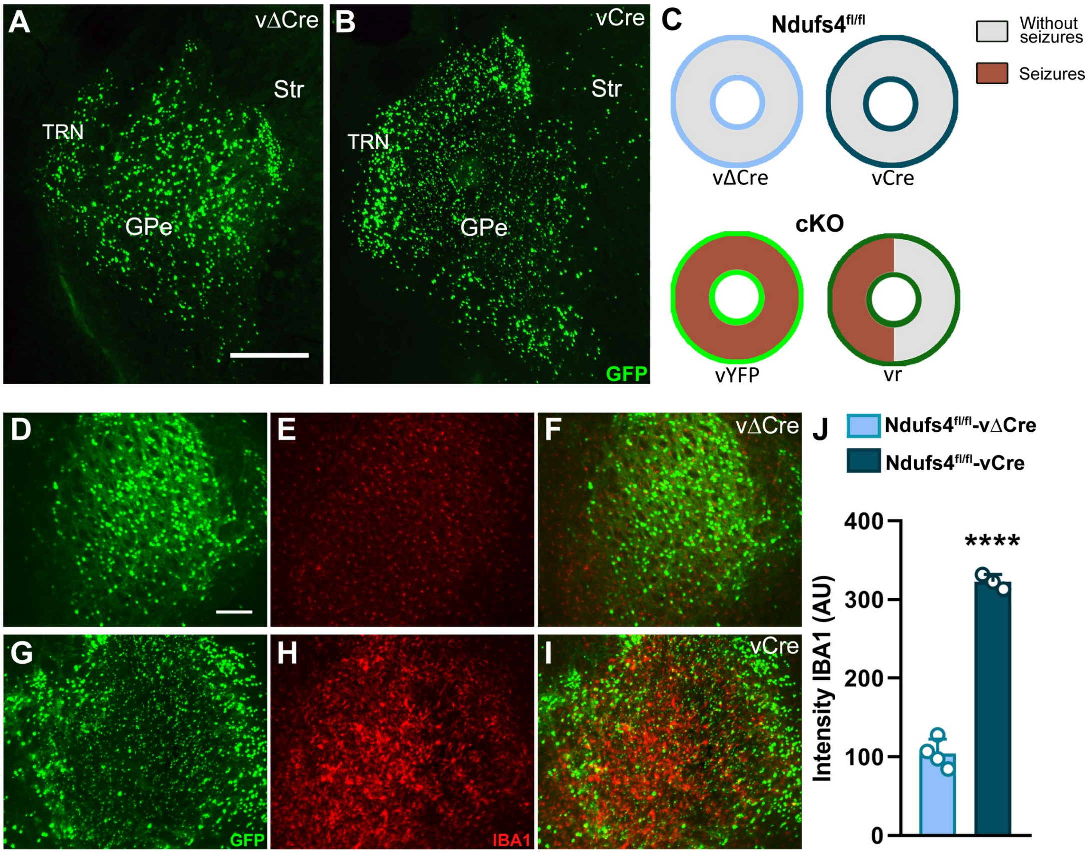

Figure S3. Effect of acute *Ndufs4* deletion in the GPe on seizure incidence and microglial reactivity. A-B) Representative images showing transduction efficiency in the GPe of the AAV1-ΔCRE·GFP (vΔCre; A) and the AAV1-CRE·GFP (vCre; B) viral vectors. **C)** Percentage of epileptic events during the induction protocol in Ndufs4$^{fl/fl}$ and cKO mice. Ndufs4$^{fl/fl}$ mice with vΔCre or vCre (n=6); cKO mice with vYFP (n=5) or vr (n=6); Scale bar = 400 µm. RTN: Reticular thalamic nucleus, GPe: Globus pallidus externus, Str: striatum. **D-J)** Immunofluorescence analysis for IBA-1 (red) and GFP (green) showing microgliosis following Cre-mediated *Ndufs4* deletion in neurons of the GPe of Ndufs4$^{fl/fl}$ mice. Panels **D** and **G** show localization of the GFP fluorescent protein from the AAV1-ΔCRE·GFP or the AAV1-CRE·GFP viral vectors, respectively. Panels **E** and **H** show expression of the microglial marker IBA1 in the GPe of Ndufs4$^{fl/fl}$ mice transduced with AAV1-ΔCRE·GFP or AAV1-CRE·GFP, respectively. Panels **F** and **I** show the overlay of both stainings. Graph in J shows a quantification of IBA1 intensity in Ndufs4$^{fl/fl}$ mice injected with AAV1-ΔCRE·GFP (Ndufs4$^{fl/fl}$-vΔCre; n=4) or AAV1-CRE·GFP (Ndufs4$^{fl/fl}$-vCre; n=3). **** $p<0.0001$, unpaired t-test.

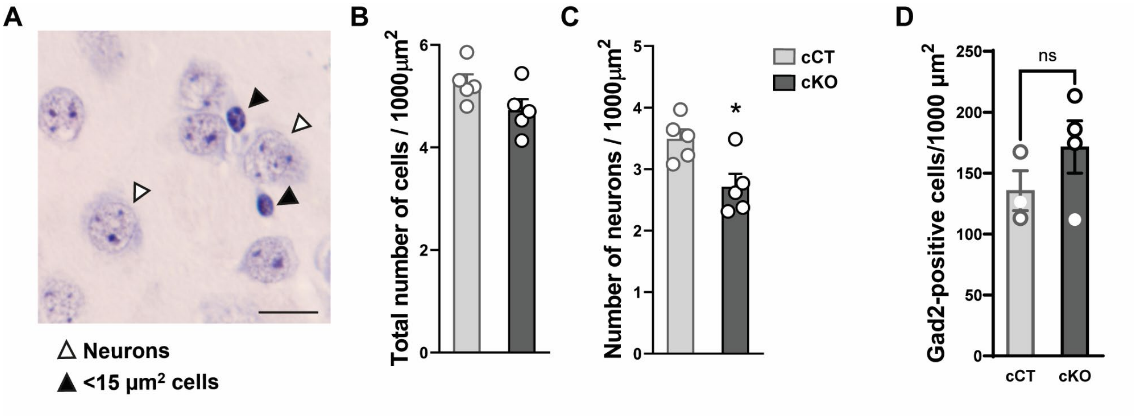

Figure S4. Neuronal density in the GPe and TRN of cKO mice A) Representative image of hematoxylin-stained section of the rostral GPe showing neurons and smaller cells (<15 µm^2), highlighted by arrows. Scale bar = 15 µm. **B)** Quantification of total cell density (number of cells / area) in the rostral GPe (Bregma –0.22 mm to -0.46 mm) of cCT and cKO mice (n=5 per group). **C)** Quantification of neuronal density (number of neurons / area) in the same region and animals. * $p<0.05$, unpaired t-test. **D)** Quantification of the total number of Gad2-expressing neurons in the TRN of cKO anc cCT mice (from Bregma –0.22 mm to –0.46 mm). n=3-4 per group.

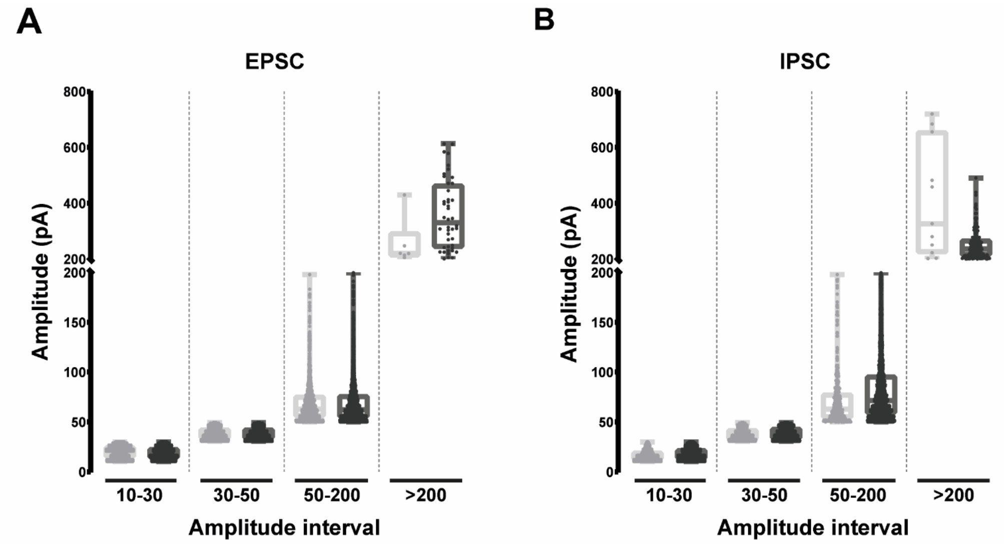

Figure S5. Increased number of high-amplitude spontaneous events in the GPe of cKO mice. Amplitude distribution of EPSCs **(A)** and IPSCs **(B)** grouped by amplitude range (n = 17 cells, 6 mice per group). p < 0,0001, Mann-Whitney test
