# Supplementary figures and images for: Dysfunctional LHX6 pallido-subthalamic projections mediate epileptic events in a mouse model of Leigh syndrome
Source: J Clin Invest. 2025 Oct 2;135(23):e187571. doi: 10.1172/JCI187571 (PMC12646652; doi:10.1172/JCI187571)

## Anti-NDUFS4

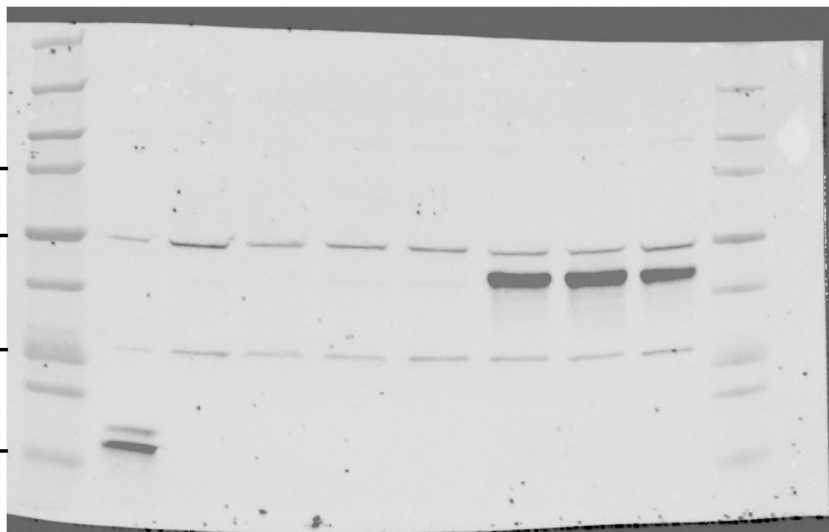

## Anti- $\beta$ TUBULIN

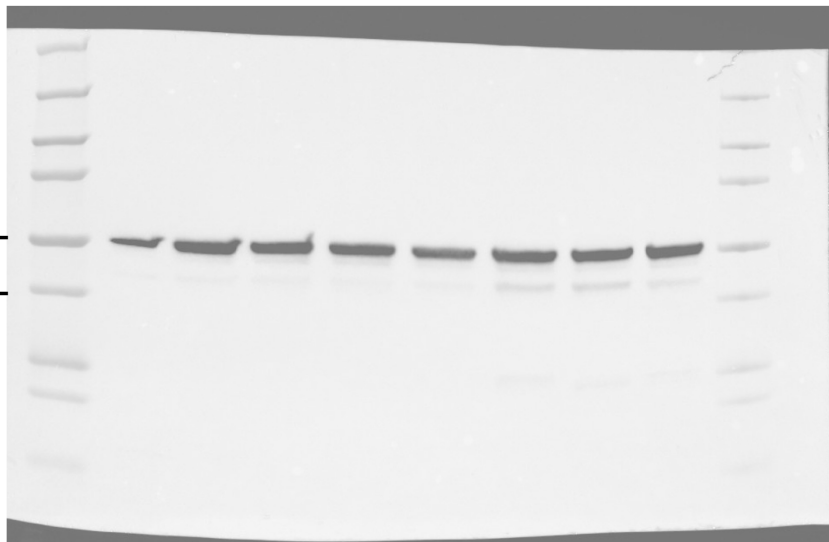

Supplement: Unedited blot and gel images [file jci-135-187571-s151.pdf]
